# Occurrence, Dissipation and Risk Assessment of Widespread Pesticides and Their Metabolites in Pomegranates

**DOI:** 10.3390/foods14223901

**Published:** 2025-11-14

**Authors:** Yuxiao Zhu, Rumei Li, Tongjin Liu, Ruijuan Li, Feng Fang, Hui Liang

**Affiliations:** 1 Institute of Plant Protection, Shandong Academy of Agricultural Sciences, Jinan 250100, China; sdgtzyx@163.com (Y.Z.); rumeili0815@163.com (R.L.); tongjinliu2024@163.com (T.L.); ruijuanli@163.com (R.L.); 2 National Center for Agricultural Biosafety Sciences Shandong Sub-Center, Jinan 250100, China; 3 Shandong Key Laboratory for Green Prevention and Control of Agricultural Pests, Jinan 250100, China

**Keywords:** residue behavior, dietary risk, pomegranate, nationwide trials

## Abstract

This study investigated the occurrence, dissipation, and dietary risks of four pesticides (difenoconazole (DIF), prochloraz (PRO), spinosad (SPI), dinotefuran (DIN)) and their metabolites in pomegranates through nationwide field trials across six Chinese production regions. Results indicated that SPI and DIN dissipated within 7–14 days, while DIF and PRO had longer half-lives (4.91–12.90 days). All pesticide residues remained confined to peels without penetrating arils. Terminal residues were below China’s MRLs. While deterministic and probabilistic risk assessments confirmed acceptable acute and chronic risks from pomegranate consumption alone (%ARfD: 0.09–17.66%; %ADI: 0.21–17.65%), comprehensive multi-crop dietary assessment revealed unacceptable chronic exposure risks for children aged under 11 years (%ADI: 56.1–155%). The non-carcinogenic risk (%HQ) for PRO from pomegranate consumption was 2.1–21.0%, indicating acceptable safety. The study provides data for safe pesticide use while highlighting the urgent need to protect vulnerable pediatric populations from cumulative pesticide exposure across multiple food sources.

## 1. Introduction

The pomegranate (*Punica granatum* L.), a member of the Punicaceae family, is native to regions such as Iran, China, and Afghanistan [1]. The pomegranate is recognized as a “superfruit” due to its exceptional nutritional and medicinal properties, and it has held historical significance in human diets and traditional medicine for centuries [2]. Extensive studies have linked various parts of the pomegranate, including its peel, arils, and leaves, to a range of biological activities and medicinal effects, such as antimicrobial, antioxidant, anthelmintic, and anti-inflammatory properties [3]. Due to the distinctive taste and numerous health benefits, pomegranates have garnered worldwide attention. Global annual pomegranate production in 2021 was estimated at 8.1 million tons, with China being a major producer, accounting for 20% of the world’s share (1.70 million tons) [4]. However, pomegranates are susceptible to various diseases and pests during growth, ripening, and post-harvest storage, including leaf spot, anthracnose, apolygus lucorum, and aphids, which can cause substantial yield losses and severe economic impacts without effective control measures [5]. Consequently, chemical control strategies, particularly the use of pesticides, are widely employed as the primary approach. Nevertheless, pesticide use inevitably leads to residue contamination concerns. Therefore, to ensure agricultural product quality safety and human health, multiple studies have been conducted on pesticide residue analysis in pomegranates. For instance, a simultaneous determination method for pesticide residues in pomegranate whole fruit and arils using ultra-high performance liquid chromatography-tandem mass spectrometry (UHPLC-MS/MS) was developed, establishing a 25 min analytical procedure for 74 pesticides [6]. Residues of 316 pesticides were monitored in 342 pomegranate samples from Turkey using UHPLC-MS/MS. Pesticide residues were detected in 72.8% of the samples, with 40 different pesticides identified. Notably, acetamiprid posed an unacceptable acute risk to children [4]. The degradation period for indoxacarb and thiamethoxam to reach levels below the Maximum Residue Limit (MRL) in pomegranate was 31.3–34 days and 46.4–51.6 days, respectively [7]. This demonstrates that certain pesticides exhibit considerable persistence in pomegranates. Systemic pesticides may migrate to the inner portions of fruit tissues, where they remain unaffected by environmental parameters, and residues within the fruit tissues dissipate primarily through growth dilution effects, thus resulting in greater persistence [8]. Given the global significance of pomegranates as nutrient-dense “superfruits” and the concerning pesticide residues that pose potential food safety hazards, systematic investigation of these residue levels constitutes a critical component of food safety assurance.

According to the China Pesticide Information Network, DIF, PRO, SPI, and DIN (Figure S1) are widely used pesticides for efficient prevention and control of pomegranate pests and diseases. These four pesticides exhibit distinct modes of action: DIF and PRO are triazole and imidazole fungicides, respectively, both inhibiting ergosterol biosynthesis in fungal cell membranes by targeting the 14α-demethylase enzyme (CYP51); SPI, a macrocyclic lactone biopesticide, activates nicotinic acetylcholine receptors (nAChRs) in insect nervous systems, causing persistent neuronal excitation; while DIN, a neonicotinoid insecticide, acts as an agonist of insect nAChRs, leading to neural overstimulation [9,10,11,12]. Several studies have investigated the residues and dissipation of these pesticides in various crops and confirmed that these pesticide residues tend to exhibit long persistence after field application, consequently posing high dietary risks. For example, dietary risk from DIF in peppers, with risk quotients (RQs) of up to 140%, was demonstrated [13]. Among 745 seasonal fruit samples, DIF was detected in 34.4% of samples and classified as a high-risk compound [14]. In 123 samples from Hainan, PRO was detected in 38.21% of samples and was assessed as a medium-risk pesticide [15]. DIN was detected in all 19 types of crop samples (fruits, vegetables, and tea) from farmland in nine prefectural cities in Zhejiang Province [16]. Numerous studies have indicated that pesticide residues can be transmitted and accumulated along the food chain, thereby threatening human health. Indeed, DIF, DIN, and PRO residues have been observed in urine and hair samples from various population groups, including children, women of reproductive age, and adults [17,18,19]. These compounds have been confirmed to pose potential risks to mammals. Specifically, studies have revealed that DIF, PRO, SPI, and DIN cause multiple toxicological effects, including developmental toxicity, reproductive toxicity, endocrine disruption effects, neurotoxicity, and transgenerational toxicity [18,20,21,22,23]. Furthermore, it is worth noting that the metabolites of PRO containing the 2,4,6-trichlorophenol moiety are toxic, mutagenic, and carcinogenic pollutants [9,24]. Currently, the residue definition of prochloraz is the sum of prochloraz and its metabolites containing the 2,4,6-trichlorophenol moiety in China [25]. The residue definition of DIN includes the parent compound and its two main metabolites (DN and UF), with these metabolites showing toxicity comparable to that of dinotefuran [26,27]. Therefore, research on DIF, PRO, SPI, DIN, and metabolites in crops is urgently needed due to their widespread residues in crops, confirmed human exposure, and health risks.

Given the global importance of pomegranates as nutritious “superfruits” and their economic value, this study focuses on four pesticides (DIF, PRO, DIN, SPI) widely used in their production process. These pesticides exhibit long persistence in crops and have been confirmed to pose health risks. However, the occurrence, dissipation, and risks of these four pesticides and their metabolites in pomegranates remain unknown. Therefore, this study aimed to (1) develop a sensitive and rapid method using UHPLC-MS/MS for the analysis of four pesticides and their metabolites in pomegranates; (2) determine the occurrence, dissipation, and concentration variation in four pesticides in pomegranates through a nationwide field experiment system, and compare MRLs in China and elsewhere; (3) systematically evaluate the acute and chronic dietary risks of four pesticides across different populations using deterministic and probabilistic models, and assess the non-carcinogenic effects of PRO. This research provides a robust scientific basis for rational pesticide use and monitoring in pomegranates, ensuring food safety while protecting public health—particularly vulnerable populations such as children.

## 2. Materials and Methods

### 2.1. Chemicals and Reagents

DIF standard (C_19_H_17_Cl_2_N_3_O_3_, CAS No. 119446-68-3, 98.5% purity), PRO standard (C_15_H_16_Cl_3_N_3_O_2_, CAS No. 67747-09-5, 99.5% purity), 2,4,6-Trichlorophenol (C_6_H_3_Cl_3_O, CAS No. 88-06-2, 97.5% purity), SPI (C_41_H_65_NO_10_-SPI-A, CAS No. 131929-60-7, 95.6% purity; C_42_H_67_NO_10_-SPI-D, CAS No. 131929-63-0, 3.4% purity), DIN (C_7_H_14_N_4_O_3_, CAS No. 165252-70-0, 99.7% purity), UF (C_7_H_14_N_2_O_2_, CAS No. 457614-34-5, 99.0% purity), DN (C_7_H_15_N_3_O, CAS No. 457614-32-3, 99.2% purity) were provided by Beijing Manhage Biotechnology Co. (Beijing, China). All reference standards were dissolved in acetonitrile/petroleum ether and stored at −20 °C until analysis. High-performance liquid chromatography (HPLC) grade acetonitrile and formic acid were sourced from Fisher Scientific (Geel, Belgium) and Sigma-Aldrich Chemical Co., Ltd. (Darmstadt, Germany), respectively. Analytical grade sodium chloride, anhydrous sodium sulfate, pyridine hydrochloride, petroleum ether and anhydrous magnesium sulfate were obtained from Sinopharm Chemical Reagent (Shanghai, China). Primary secondary amine (PSA), octadecylsilane (C18, 40 μm), and nylon filters (0.22 μm) were acquired from Agilent Technologies (Beijing, China).

### 2.2. Field Trials and Sample Collection

Following the OECD Chemical Testing Guideline No. 509, field trials were established in six provinces across China’s primary pomegranate cultivation areas (Table S1). Each experimental site consisted of one treatment plot and one control plot, with each plot containing at least four pomegranate trees. DIF, PRO, SPI and DIN were applied at their maximum recommended application rates and dosages. Detailed information on pesticide applications is provided in Table S2. For terminal residue determination, pomegranate samples were collected at 14-, 21-, and 28-day pre-harvest intervals (PHI) in six planting provinces (Trials #1–#6). For dissipation kinetics assessment, samples were collected at 0, 7, 14, 21, and 28 days after application in three selected provinces (Trial #1, Trial #2, and Trial #5). Each sample consisted of 12 fruits collected using scissors from at least 4 plants, sampling from different orientations and positions (top, middle, bottom, inner, and outer parts), with a minimum weight of 2 kg. All collected samples were chopped into pieces, reduced to subsamples through the quartering method, and subsequently stored at –20 °C under dark conditions. Detailed information regarding growth stage and climatic conditions for each trial site is presented in Table S1.

### 2.3. Analytical Procedures

For the detection of DIF, SPI, DIN and metabolites (UF and DN), a modified QuEChERS method was employed. Pomegranate arils or whole pomegranate samples were precisely measured (10.0 ± 0.01 g) and transferred into separate 50 mL centrifuge tubes with caps. A volume of 10 mL acetonitrile with 1% formic acid was added to each tube. The samples were vigorously oscillated for 10 min. Following this, 1 g of NaCl and 4 g of MgSO_4_ were added to each tube. The mixture was subsequently vortexed for 5 min and then centrifuged at 4000 rpm for 5 min. Then, 1.5 mL of supernatant was collected and placed into 2 mL tubes pre-loaded with 50 mg PSA, 50 mg C18, and 150 mg MgSO_4_. These tubes were vortexed for 1 min before being centrifuged at 9000 rpm for 5 min. The resulting extracts were passed through 0.22-μm nylon membrane syringe filters and collected directly in autosampler vials, ready for UHPLC-MS/MS determination.

For prochloraz, a chemical derivatization method was employed to determine the total residue (sum of prochloraz and its metabolites containing the 2,4,6-trichlorophenol moiety) as required by the Chinese residue definition. Both the parent compound and its metabolites were converted to 2,4,6-trichlorophenol for quantification. A total of 10.0 g of pomegranate arils or whole pomegranate samples were weighed into a 50 mL centrifuge tube, followed by the addition of 20 mL acetonitrile. The mixture was vortexed for 5 min, then 4 g of sodium chloride was added and the mixture was vortexed for 2 min. After centrifugation at 8000 rpm for 5 min, 10 mL of the supernatant was transferred to a round-bottom flask and concentrated to near dryness. To the round-bottom flask, 8 g of pyridinium hydrochloride and a few boiling stones were added. A condenser was attached and the flask was placed in an oil bath. The top of the condenser was sealed with cotton wool, and the mixture was heated at 220 °C for 1 h. After the reaction was complete, the flask was allowed to cool. The round-bottom flask was rinsed with several portions of distilled water (40 mL total), and the contents were transferred to a separatory funnel for purification. The aqueous phase was discarded, and the petroleum ether extract was dried over anhydrous sodium sulfate and transferred to a round-bottom flask. The extract was concentrated to near dryness and reconstituted in 5 mL with petroleum ether. The final extract was filtered through a 0.22 μm membrane filter and analyzed by gas chromatography.

Analysis of DIF, SPI, DIN and metabolites (UF and DN) was conducted using an ACQUITY UHPLC system coupled with an XEVO TQD quadrupole mass spectrometer equipped with an electrospray ionization source (ESI) (Waters, Milford, MA, USA). Chromatographic separation of DIF, SPI, DIN and metabolites (UF and DN) was achieved using a BEH C18 column (2.1 mm × 50 mm, 1.7 μm particle size, Waters Corporation). The mobile phases comprised methanol (phase A) and 0.1% formic acid solution with 5 mmol/L ammonium acetate (phase B). Gradient elution was conducted as follows (0.3 mL/min): 0 min, 10% A; 1.2 min, 90% A; 1.2–1.5 min, 80% A; 1.5–3.5 min, 80% A; 3.5–3.6 min, 10% A; 3.6–7.0 min, 10% A. The chromatographic conditions were optimized with column temperature of 40 °C and injection volume of 2 μL. Mass spectrometric detection of DIF, SPI, DIN and metabolites (UF and DN) was optimized in positive ionization mode with the following parameters: capillary voltage of 2.5 kV, source temperature of 150 °C, and desolvation temperature of 350 °C. Cone and desolvation gas flows (N_2_) were maintained at 50 and 650 L/h, respectively. Multiple reaction monitoring (MRM) transitions were established for DIF, SPI, DIN and metabolites (UF and DN) (Table S3).

Analysis of 2,4,6-trichlorophenol was performed using an Agilent 7890B gas chromatograph equipped with an electron capture detector (ECD) and a BP×608 capillary column (25 m × 0.32 mm i.d., 0.4 μm film thickness). The instrumental conditions were set as follows: injector temperature at 250 °C, detector temperature at 280 °C, and oven temperature programmed from an initial temperature of 80 °C (held for 0 min), ramped to 200 °C at 10 °C/min (held for 1 min), then ramped to 280 °C at 15 °C/min (held for 5 min), using nitrogen as carrier gas at a flow rate of 1.5 mL/min with splitless injection of 1 μL.

### 2.4. Dietary Risk Assessment

The deterministic approach for risk evaluation was originally established by the Joint FAO/WHO Expert Meetings on Pesticide Residues (JMPR) and serves as a methodology for analyzing both acute and chronic dietary exposure assessment.

(1) IESTI = (LP × HR × v)/bw

(2) %ARfD = (IESTI/ARfD) × 100%

Acute dietary consumption of whole pomegranate fruit was performed utilizing the International Estimated Short-term Intake (IESTI) framework. In this formula, HR represents the highest residue levels detected in pomegranate crop samples. LP (g/person) denotes the large portion consumption of whole pomegranate (97.5th percentile among consumers), and bw (kg) represents the average body weight of the target population subgroup. The variability factor v is set at 3. The toxicological thresholds of the acute reference dose (ARfD) serve as critical standards in dietary risk assessment studies. Short-term dietary exposure risk is considered acceptable when the %ARfD remains below 100%. Conversely, when the %ARfD exceeds 100%, the consumption of crops containing pesticide residues poses a potential short-term exposure hazard.

(3) $$NEDI=\sum \frac{\left(STMR_{i}\times F_{i}\right)}{bw}$$

(4) $$\%ADI=\frac{NEDI}{ADI}\times 100\%$$

Chronic dietary exposure assessment is related to the estimated daily intake (NEDI, mg/kg bw) and acceptable daily intake (ADI, mg/kg bw). The long-term exposure risk from pesticide residues was quantified using Equations (3) and (4). In this assessment model, STMR_i_ denotes the supervised trial median residue concentration determined from whole pomegranate samples, whereas F_i_ (g/d) represents the mean daily consumption data of whole pomegranate for the specific population. The chronic dietary risk is expressed as a percentage of the acceptable daily intake (%ADI), where risk levels are considered toxicologically acceptable when %ADI values do not exceed 100%.

(5) $$y_{ij}=\frac{\sum_{k=1}^{p}\left(x_{ijk}\times c_{ijk}\right)}{bw_{i}}$$

(6) $$\%ADI=\frac{y_{ij}}{ADI}\times 100\%$$

Probabilistic model represents an established analytical approach extensively utilized for chronic dietary exposure assessment of pesticide residues across regulatory authorities in the European Union and United States. The probabilistic model enhances risk characterization accuracy for human dietary exposure to pesticide residues, following Equations (5) and (6). y_ij_ represents the compound intake by Group i on Day j, whereas *p* denotes the total count of examined food items. x_ijk_ quantifies whole pomegranate consumption rates for demographic group i on day j, while c_ijk_ characterizes the pesticide concentration in whole pomegranate ingested by Group i on Day j. B_wi_ is the body weight of Group i.

(7) ADD=(C×IR×EF×ED)/(bw×AT)

(8) %HQ=ADD/RfD×100%

where ADD is the mean daily dietary dose (mg/kg·day), EF is the exposure frequency (365 days/year), ED refers to the exposure duration (70 years), AT represents the averaging time (in days), defined as ED × 365, C represents the mean pesticide residue concentration (mg/kg), and IR denotes the daily pomegranate intake (mg/day). The reference dose (RfD) for the pesticide is used to evaluate potential non-carcinogenic risks. %HQ below 100% indicates that adverse non-carcinogenic effects are unlikely in the exposed population, while a value equal to or exceeding 100% suggests possible adverse health effects.

### 2.5. Data Analysis

The matrix effect (ME) refers to the impact of co-eluting substances from pomegranate on the detector response and was computed according to the formula below:

(9) $$ME\%=\left(\frac{K_{1}}{K_{2}}-1\right)\times 100\%$$

where ME is the matrix effect, k_1_ indicates the slope of the matrix-based standard curve, and k_2_ represents the slope of the solvent-based standard curve. Values of ME% ≥ 10% signify pronounced matrix signal enhancement, whereas ME% ≤ −10% indicates marked matrix signal suppression. The matrix influence becomes insignificant within the range of −10% < ME% < 10% [28].

Measurement uncertainty was assessed following EURACHEM/CITAC guidelines [29]. Accuracy uncertainty (Ua, Equation (10)) and precision uncertainty (Up, Equation (11)) were combined to calculate the expanded uncertainty (U, Equation (12)).

(10) $$\mathrm{Ua}=\mathrm{SDr}/\sqrt{n}$$

SDr is the standard deviation of recovery for replicate measurements in the intraday precision test.

(11) $$\mathrm{Up}=\mathrm{SDR}/\sqrt{n}$$

SD_R_ is the standard deviation of repeatability for replicate measurements in the interday precision test.

(12) $$U=K\times \sqrt{{Ua}^{2}+{Up}^{2}}$$

K is the coverage factor of 2, with a confidence level of 95%.

The dissipation behavior of four pesticides in pomegranates was assessed using diverse kinetic modeling approaches. Half-life calculations were performed by employing different mathematical models: the single first-order (SFO) model (Formulas (13) and (14)), the double first-order in parallel (DFOP) model (Formula (15)), and the first-order multiple compartments (FOMC) model (Formula (16)).

(13) $$C={\;C}_{0}\times e^{-kt}$$

(14) $$T_{1/2}=\frac{ln2}{k}=\frac{0.693}{k}$$

where C_0_ and C represent the pesticide concentrations at the initial time point and at time t following treatment, respectively. The half-life corresponds to the time for the pesticide level to decline to 50% of its original concentration.

(15) $$C_{t}={\;C}_{0}\times \left({g\times e}^{-k_{1}\times \;t}+\left(1-g\right)e^{{-k}_{2}\times t}\right)$$

where g denotes the proportion of C_0_ present in the solution phase, k_1_ and k_2_ correspond to the kinetic constants for the rapid and gradual sub-reactions, respectively [25].

(16) $$C_{t}=\frac{C_{0}}{{\left(\frac{t}{\beta }+1\right)}^{\alpha }}$$

where α and β denote parameters of the underlying probability density function Γ [26].

## 3. Results

### 3.1. Method Validation

Considering that certain systemic pesticides can penetrate from pomegranate peel into edible portions, analytical methods were developed and validated for both pomegranate arils and whole fruit matrices. Two distinct sample preparation approaches were employed: (1) a modified QuEChERS method coupled with UHPLC-MS/MS for DIF, SPI, DIN, and their metabolites (UF and DN), and (2) a conversion method with GC-ECD for PRO and its metabolites (expressed as 2,4,6-trichlorophenol equivalents), in accordance with JMPR dietary risk assessment definitions. Both methods were applied to each matrix type (arils and whole fruit) to enable comprehensive residue distribution analysis. The specificity, linearity, limit of quantitation (LOQ), matrix effect (ME%), precision (intra-day and inter-day repeatability), and accuracy were comprehensively validated according to the analytical quality control criteria of SANTE/11312/2021 [30]. The method validation results for the seven analytes in pomegranate are summarized in Table 1. Blank assays of pomegranate arils and whole pomegranate matrices were conducted to verify the specificity of the described method, with no detectable interference observed at the retention times of DIF (2.33 min), 2,4,6-trichlorophenol (9.54 min), SPI-A (0.78 min), SPI-D (0.99 min), DIN (0.45 min), UF (0.45 min), and DN (0.42 min) (Figure S2). Considering the matrix effects of UHPLC-MS/MS, linearity was verified using both solvent- and matrix-matched standard calibration curves with concentrations ranging from 0.01 to 1 mg/L. Excellent linearity was achieved for all analytes, with correlation coefficients (R^2^) ranging from 0.9908 to 0.9996. The matrix effects for DIF, SPI-A, SPI-D, DIN, UF, and DN in pomegranate arils and whole pomegranate ranged from −6.3% to 17.5%. Therefore, external matrix-matched standards were selected for quantitative analysis of these six compounds to minimize matrix effects. PRO was converted to 2,4,6-trichlorophenol and quantified by GC-ECD. Matrix effects in the GC-ECD analysis of 2,4,6-trichlorophenol were evaluated and found to be negligible (Table 1). Solvent-based calibration using petroleum ether was employed, yielding excellent linearity (R^2^ = 0.9983) over the concentration range of 0.03–3 mg/L. Based on established MRLs, appropriate spiked concentrations were determined, with LOQ set as the lowest spiked level. The LOQs for DIF, SPI, DIN, UF, and DN were 0.02 mg/kg, while that for 2,4,6-trichlorophenol was 0.05 mg/kg. The accuracy of the method was validated through recovery tests at three concentration levels with five replicates each. Average recoveries for the seven analytes ranged from 73.00% to 115.93%. The precision and repeatability of the method were assessed through the relative standard deviation (RSD) of recovery on the same day (intraday, n = 5) and on three different days (interday, n = 3). The RSDs of intraday and interday precision were 0.6–12.0% and 1.3–9.1%, respectively. The expanded measurement uncertainty ranged from 2.7 to 15.1%. According to the European Commission, the acceptable expanded uncertainty is within 44% below 100 μg/kg and within 32% at 1 mg/kg [31]. Overall, these results demonstrate that the developed method is suitable and reliable for the determination of the seven target analytes in pomegranate samples.

### 3.2. Occurrence and Dissipation of Four Pesticides in Both Pomegranate Arils and Whole Pomegranate Matrices

As shown in Table S4, the initial deposition concentrations of the four pesticides in Trial #5 were 1.3–5.4 times and 1.5–8.5 times higher than those in Trial #1 and Trial #2, respectively. Consequently, the deposition of all four pesticides in Trial #5 was significantly higher than in Trials #1 and Trial #2. The reason for this substantial difference is related to the morphological characteristics of the crop itself: the cultivar planted in Trial #5 (Hong Hua Yu Shi Zi) possesses larger leaf and fruit surface areas and a more open canopy structure, which therefore enables more efficient interception of spray droplets [28]. There are notable differences in pesticide deposition efficiency among different pomegranate cultivars. The fastest dissipation was observed for SPI, with complete dissipation being achieved within 7 days, followed by DIN, for which complete dissipation was observed within 14 days, and no metabolites of DIN (DN and UF) were detected. For DIF and PRO, 28-day dissipation rates of 85–91% and 85–93% were observed, respectively. Due to the rapid dissipation of DIN and SPI, their dissipation half-lives were not calculated. Through observation of DIF and PRO dissipation, distinct dissipation rates were identified across different time periods. For instance, a rapid dissipation rate (51–58%) was observed for DIF during the first seven days, followed by a subsequent slow dissipation phase. Therefore, three different dissipation kinetic models—SFO, DFOP, and FOMC—were employed to fit the data and estimate half-lives. Table 2 provides the calculated dissipation half-lives derived from the three modeling approaches. The optimal model was selected based on the highest coefficient of determination (R^2^), the lowest chi-square (χ^2^) error, and the residual distribution patterns [32]. For PRO dissipation, the SFO model proved to be the optimal fitting approach (R^2^ = 0.9394–0.9932; χ^2^ error = 3.82–13.2%) compared to DFOP (R^2^ = 0.9395–0.9932; χ^2^ error = 5.45–18.9%) and FOMC (R^2^ = 0.9394–0.9932; χ^2^ error = 4.37–15.1%), calculating dissipation half-lives ranging from 9.61 to 12.9 days. For DIF dissipation, the DFOP model demonstrated superior fitting (R^2^ = 0.9814–0.9996; χ^2^ error = 1.24–11.5%) in determining dissipation half-lives (4.91–11.4 days) compared to the SFO (R^2^ = 0.9469–0.9947; χ^2^ error = 13.15–13.7%) and FOMC (R^2^ = 0.9691–0.9992; χ^2^ error = 1.34–11.8%) models. Overall, the four pesticides ranked by decreasing dissipation rates were SPI > DIN > PRO > DIF, which may be related to the physical and chemical properties of the pesticides. For example, under simulated light irradiation for 15 days, DIF exhibited less than 10% dissipation (JMPR, 2007), whereas both SPI (JMPR, 2001) and DIN (JMPR, 2012) demonstrated photolysis half-lives of less than 1 day—findings that align with the observed dissipation regularity Additionally, dissipation half-lives were also related to climatic temperature. The dissipation half-lives of DIF and PRO in Trial #1 (11.7 and 6.68 days, respectively) and Trial #2 (12.9 and 11.4 days, respectively) were 1.2–1.3 times and 1.3–2.32 times longer than in Trial #5 (9.6 and 4.91 days, respectively), respectively. Investigation revealed that during the experimental period, precipitation and temperature in Trial #5 were significantly higher than those in Trial #1 and Trial #2, and consequently, high temperature and humidity conditions could accelerate compound evaporation and dissipation [33]. For pomegranate arils, no pesticides were detected, indicating that pesticide residues remained on the pomegranate rind without movement to the aril. These findings demonstrated that pesticides are primarily distributed on the pomegranate peel without penetrating into the internal tissues. This distribution is consistent with results obtained for eleven other pesticides, including acephate, thiamethoxam, and tebuconazole, which were detected only in whole pomegranates but not in arils [6]. Similarly, buprofezin and dimethoate residues were confined exclusively to the peel without translocation to internal parts. Although imidacloprid was detected in internal fruit tissues, concentrations remained below the LOQ [34]. In brief, pesticide dissipation is closely related to the physicochemical properties of compounds, crop varieties, and environmental conditions.

### 3.3. Terminal Levels and MRL Comparison of DIF, PRO, SPI, DIN and Their Metabolites in Pomegranates

**Figure 1:**
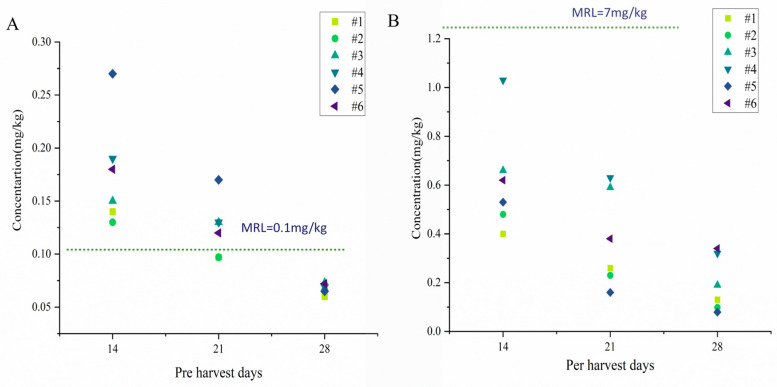
Terminal levels of DIF (**A**) and PRO (**B**) in pomegranate with PHI of 14 d, 21 d, and 28 d.

The terminal concentrations of DIF, PRO, SPI, and DIN and their metabolites in pomegranates were investigated at six representative sites across southern and northern climatic zones of China. No pesticides were detected in the pomegranate arils, which is consistent with previous reports indicating that in pomegranates, the major part of pesticides remained in the outer peel, with limited movement to the inner peel [35]. The terminal concentrations of DIF and PRO ranged from 0.060 to 0.27 mg/kg and from 0.079 to 1.03 mg/kg in the whole fruit, respectively. Furthermore, SPI, DIN, and DIN’s metabolites (DN and UF) were not detected in the whole fruit, consistent with DIN dissipation studies in eggplant where both metabolites DN and UF remained below the LOQ [36]. The concentrations of both DIF and PRO decreased progressively over time (Figure 1), following typical pesticide dissipation patterns in crops. According to MRLs of 0.1 mg/kg for DIF and 7 mg/kg for PRO in China, the recommended pre-harvest intervals (PHIs) are 28 days and 14 days, respectively. The supervised trials median residue (STMR) and highest residue (HR) values for DIF in pomegranates were determined to be 0.068 mg/kg and 0.073 mg/kg, respectively, while those for PRO were 0.58 mg/kg and 1.03 mg/kg, respectively. As one of the world’s largest pomegranate producers, China’s pesticide residue control standards directly determine its competitive position in export trade. Currently, the MRLs established by the Codex Alimentarius Commission (CAC) and European Union for DIF in pomegranates are 0.15 mg/kg and 0.1 mg/kg, respectively. Since these international standards are equal to or higher than China’s domestic MRL, Chinese pomegranate exports to these countries are unlikely to encounter trade barriers related to DIF residues. However, the European Union has established a significantly lower MRL of 0.3 mg/kg for PRO in pomegranates compared to China’s 7 mg/kg. Consequently, Chinese pomegranate exports to the EU market require strict monitoring to prevent trade barriers caused by excessive pesticide residues exceeding this threshold. For SPI and DIN, although residues were below LOQ in this study, international trade implications should be closely monitored, as China currently lacks MRLs for these pesticides in pomegranates. This regulatory gap poses potential trade risks and exposure concerns. This study provides a theoretical basis and scientific reference for establishing MRLs of SPI and DIN in pomegranates, with recommended values of 0.05 mg/kg and 0.2 mg/kg, respectively, to mitigate potential exposure risks and facilitate international trade.

### 3.4. Dietary Risk Assessment of DIF and PRO Using Deterministic and Probabilistic Models

#### 3.4.1. Acute Dietary Risk Assessment of DIF and PRO

**Figure 2:**
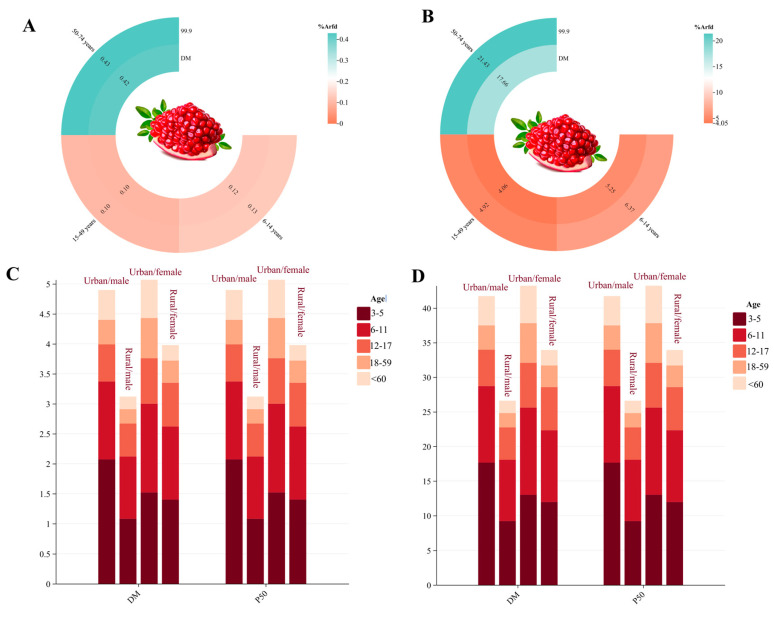
Acute dietary risk assessment of DIF (**A**) and PRO (**B**) in pomegranates by deterministic and probabilistic models, and Chronic dietary risk assessment of DIF (**C**) and PRO (**D**) in pomegranates by deterministic and probabilistic models.

The short-term risks of DIF and PRO exposure from pomegranate were evaluated based on ARfD, which were recommended as 0.3 and 0.1 mg/kg bw by the Joint Meeting on Pesticide Residues (JMPR). The large portion consumption of pomegranate (97.5th percentile of consumers) and body weights (kg) of children (6–14 years old), adults (14–59 years), and elderly (59–74 years) were obtained from FAO. Combined probabilistic and deterministic models were conducted for a more systematic assessment of DIF and PRO exposure from pomegranate. Short-term dietary risks to relevant populations were first assessed using deterministic methods, with DIF ranging from 0.09% to 0.39% and PRO ranging from 4.78% to 17.66% (Figure 2). The highest risk was observed in the elderly population aged 50–74 years, while the lowest risk was found in the 15–49 years age group. This difference was closely related to the higher per-unit body weight intake in the elderly population (5.71 g/kg bw/day for 59–74 years; 1.46–1.31 g/kg bw/day for 14–59 years; 1.54–1.69 g/kg bw/day for 6–14 years). Subsequently, probabilistic modeling was employed. The probabilistic model generated more precise values for pesticide data based on log-normal distribution and Monte Carlo resampling methods when estimating dietary exposure, thereby improving the accuracy of risk assessment [37]. For DIF, the estimated risk from the deterministic model based on HR was similar to the 99.9th percentile results from probabilistic model. However, for PRO, the estimated risk from the deterministic model based on HR was significantly lower than the 99.9th percentile results from probabilistic model (*p* < 0.05) (Figure 2). Deterministic models employ single concentration and intake levels to obtain point exposure estimates, and consequently, occasional consumption of pomegranate samples with high or low concentrations may lead to risk overestimation or underestimation [38].

#### 3.4.2. Chronic Dietary Risk Assessment of DIF and PRO

**Figure 3:**
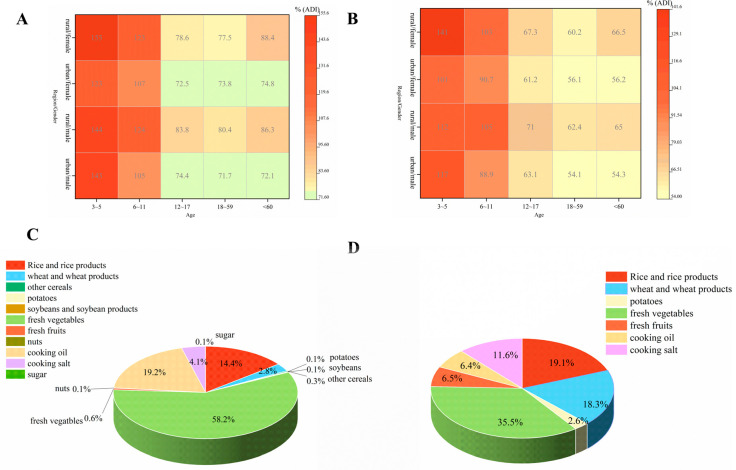
Chronic dietary risk assessment of DIF (**A**) and PRO (**B**) in registered crops by deterministic estimation, and contributions of different crop categories to dietary exposure of DIF (**C**) and PRO (**D**) for the general consumer population.

The average daily intake and body weight data were extracted from the Chinese National Nutrition and Health Survey program. Populations were stratified by gender (male, female), residence (urban, rural), and age groups (3–5, 6–11, 12–17, 18–59, >60 years). The ADI for both DIF and PRO were set at 0.01 mg/kg. As illustrated in Figure 3, the %ADI values for DIF and PRO exposure from pomegranate consumption were 0.21–1.5% and 1.79–17.65%, respectively. Assessment using both probabilistic and deterministic models demonstrated that deterministic model estimates based on STMR were similar to 50th percentile results from probabilistic model (Figure 3). However, considering that consumers typically consume multiple fruits and vegetables, a comprehensive total dietary assessment was warranted. The deterministic model was employed to evaluate DIF and PRO exposure levels across consumption of multiple crops. Following the Chinese Dietary Risk Assessment Guidelines, a total dietary risk assessment for all registered crops was executed (Table S5), with STMR values obtained from the Ministry of Agriculture and Rural Affairs and MRLs applied under the risk maximization principle when STMR data were unavailable. The assessment revealed that %ADI values of 72.1–155% and 56.1–141% were observed for DIF and PRO exposure, respectively, across all registered crops. Notably, unacceptable exposure risks were observed in children under 11 years of age who exceeded ADI thresholds for both DIF and PRO, with the most severe exposure risk (101–155%) occurring in the 3–5 years age group across all demographic groups. This age-related vulnerability was attributed to similar dietary consumption patterns as adults but significantly lower body weight, thereby resulting in higher exposure per unit body weight [39]. Furthermore, significantly higher %ADI values for both pesticides were observed in rural populations (65–155%) compared to urban populations (54.1–143%) (Figure 3), with disparities attributed to differential dietary preferences and average body weight variations between rural and urban populations [40]. Regarding crop risk contributions among the general population (18–59 years), fresh vegetables (58.2%) > cooking oil (19.2%) > rice and rice products (14.4%) were identified as the predominant risk contributors for DIF exposure (Figure 3). Similarly, comparable patterns were demonstrated for PRO exposure, with fresh vegetables (35.5%) > rice and rice products (19.1%) > wheat and wheat products (18.3%) being identified as the principal contributors (Figure 3). Fresh vegetables constituted the highest risk contributor for both DIF and PRO exposure. Urban populations (18–59 years) consumed more vegetables (urban: 275 g/day; rural: 257.2 g/day), while rural populations consumed more cooking oil (rural: 49.4 g/day; urban: 45.4 g/day), wheat (rural: 145.3 g/day; urban: 130.8 g/day) and rice (rural: 184.1 g/day; urban: 136.5 g/day), thus creating distinct exposure patterns between demographic groups. The combined effect of consumption patterns with lower average body weights (rural: 60 kg; urban: 65 kg) in rural populations significantly increased exposure risks in rural areas. Therefore, the comprehensive risk assessment demonstrated that both DIF and PRO present unacceptable dietary risks to children under 11 years of age, indicating that current MRL standards may be inadequate for protecting vulnerable pediatric populations.

### 3.5. Analysis of Non-Carcinogenic Effects of PRO Using Deterministic and Probabilistic Models

**Figure 4:**
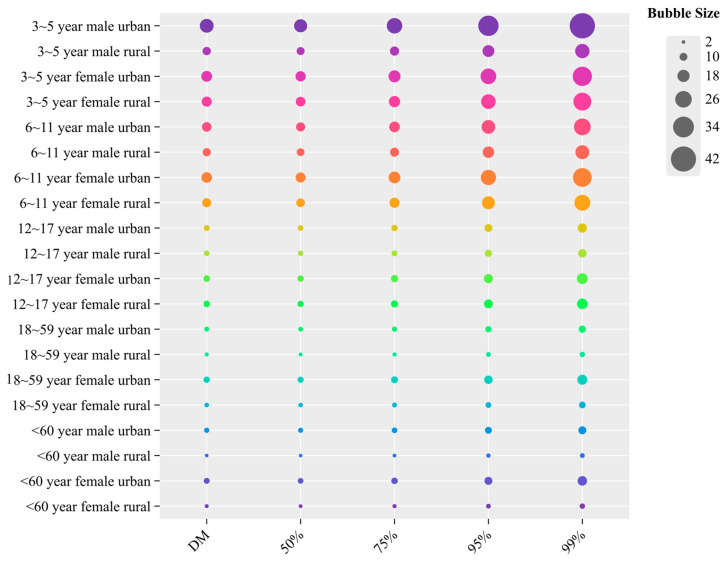
Non-carcinogenic effect effects of PRO using deterministic and probabilistic models.

According to records from the US Environmental Protection Agency’s Comprehensive Risk Information System, the non-carcinogenic reference dose (RfD) for PRO is 0.009 mg/kg while the reference dose for DIF has not yet been established in this system. Based on these reference values, to evaluate the non-carcinogenic effects of PRO on pomegranate consumption among the aforementioned populations, both deterministic and probabilistic models were applied. The deterministic risk assessment revealed that non-carcinogenic risks for PRO in pomegranates ranged from 2.1% to 21.0% (Table S6), with risk gradually decreasing with increasing age. The highest risk values were revealed among urban male children aged 3–5 years, while the lowest risk levels were exhibited by rural women over 60 years (Figure 4). Urban populations demonstrated significantly elevated risk profiles compared to rural populations (*p* < 0.05), primarily attributed to differences in intake per unit body weight across different population groups. Additionally, probabilistic models were applied to more accurately assess non-carcinogenic dietary risks across different percentiles, which gradually increased from the 50th (2.0–19.6%) to the 99.9th (4.3–42.3%) percentile (Table S6). The results indicated that even at the 99.9th percentile, the %HQ for PRO through dietary pomegranate consumption was far lower than 100%, thus representing an acceptably low risk for Chinese consumers. The 50th percentile results were most similar to the deterministic results. The probabilistic model provides a more comprehensive quantification of %HQ for PRO across multiple percentile points.

## 4. Conclusions

This study represents the first systematic investigation into the occurrence, dissipation kinetics, and dietary risk assessment of four widely used pesticides—DIF, PRO, SPI, and DIN—along with their metabolites in pomegranates. The nationwide field trials across six production regions demonstrated rapid degradation for SPI and DIN (7–14 days) versus longer half-lives for DIF and PRO (half-lives 4.91–12.90 days). All pesticide residues remain confined to pomegranate peels without translocation to aril portions. Terminal residues of DIF, PRO, SPI, DIN were below China’s MRLs, and no metabolites were detected. China currently lacks MRLs for SPI and DIN in pomegranates. Based on these findings, MRLs of 0.05 mg/kg for SPI and 0.2 mg/kg for DIN are recommended. While deterministic and probabilistic risk assessments confirmed that acute and chronic risks from pomegranate consumption alone are acceptable across all population groups, a comprehensive multi-crop exposure assessment revealed alarming chronic dietary risks for children under 11 years, with %ADI values reaching 56.1–155%. These findings reveal the inadequacy of current adult-centered risk assessment models and highlight the urgent need to protect children from pesticide exposure across multiple food sources. The pesticide residue behavior risk assessment research framework established in this study provides important theoretical foundations and technical support for scientific pesticide use, food safety regulation, and public health protection. Nevertheless, several limitations must be acknowledged. Current risk assessments are primarily based on acute and chronic toxicity data, and more research data are needed to support understanding of potential health impacts from long-term low-dose exposure, particularly effects on child development. Furthermore, the research primarily focused on single pesticide risk assessment, lacking in-depth exploration of potential synergistic or antagonistic effects when multiple pesticides are simultaneously present.

## Figures and Tables

**Table 1:** Linear regression parameters, matrix effects and mean recoveries of seven analyses in pomegranate at three spiked levels.

Compounds	Matrix	Regression Equation	R^2^	ME (%)	Spiked Levels (mg/kg)	Mean Recoveries (%)	Intraday RSD (%)	Interday RSD (%)	U (%)
DIF	MeCN	Y = 941,170X + 235	0.9957	--	--	--			
	Pomegranate aril	Y = 933,507X + 90.4	0.9964	−0.8	0.02	90	4.4	3.6	5.7
					0.1	96	1.3	2.9	3.5
					1	93	4.5	5.7	7.7
	Whole pomegranate	Y = 1,007,740X + 798	0.9969	8.0	0.02	99	1.8	1.9	2.7
					0.1	98	4.1	2.8	4.9
					1	93	1.6	8.3	9.7
2,4,6-trichlorophenol (PRO)	Petroleum ether	Y = 49,349X − 3104	0.9983	--	--	--			
	Pomegranate aril	Y = 48,874x − 3428	0.9956	−1.0	0.05	105	1.4	3.0	3.7
					0.5	108	0.9	7.1	8.2
					7	73	4.4	3.6	5.7
	Whole pomegranate	Y = 48,723x − 3041	0.9967	−1.3	0.05	91	5.2	8.5	10.9
					0.5	73	1.6	9.1	10.6
					7	80	3.5	8.7	10.5
SPI-A	MeCN	Y = 2,374,850X + 2235	0.9963	--	--	--			
	Pomegranate aril	Y = 2,311,220X + 1383	0.9966	−2.7	0.02	88	6.7	5.8	9.0
					0.3	87	4.2	7.6	9.5
					1	96	0.6	3.9	4.5
	Whole pomegranate	Y = 2,424,380X + 1811	0.9958	2.1	0.02	78	2.3	3.8	4.8
					0.3	82	2.6	3.6	4.8
					1	101	3.2	4.8	6.2
SPI-D	MeCN	Y = 4,472,690X + 27.5	0.9995	--	--	--			
	Pomegranate aril	Y = 4,392,520X − 45.8	0.9985	−1.8	0.02	79	6.9	7.4	10.5
					0.3	85	5.7	2.5	5.9
					1	95	0.6	1.3	1.6
	Whole pomegranate	Y = 4,595,450X + 1.91	0.9996	2.7	0.02	79	1.9	2.1	3.0
					0.3	78	5.0	2.4	5.3
					1	100	2.3	3.6	4.6
DIN	MeCN	Y = 548,810 X + 7618	0.9959	--	--	--			
	Pomegranate aril	Y = 514,009X + 9388	0.9956	−6.3	0.02	91	12.0	9.2	15.1
					0.2	85	7.4	7.6	11.0
					0.4	85	7.1	7.3	10.6
	Whole pomegranate	Y = 642,332X + 13,745	0.9906	17.0	0.02	99	5.1	4.8	7.2
					0.2	87	7.9	3.6	8.2
					0.4	77	7.6	9.1	12.5
UF	MeCN	Y = 228,605X + 37,826	0.9914	--	--	--			
	Pomegranate aril	Y = 221,594X + 38,766	0.9910	−3.1	0.02	94	2.5	3.6	4.7
					0.2	81	1.2	2.7	3.3
					0.4	80	1.1	3.2	3.8
	Whole pomegranate	Y =268,593.X + 49,573	0.9908	17.5	0.02	93	1.6	3.5	4.3
					0.2	82	2.7	2.4	3.7
					0.4	78	0.9	2.5	3.0
DN	MeCN	Y = 224,225X + 15,339	0.9975	--	--	--			
	Pomegranate aril	Y = 235,574X + 21,881	0.9969	5.1	0.02	98	4.5	9.8	12.0
					0.2	78	1.9	4.9	5.9
					0.4	86	2.4	5.2	6.4
	Whole pomegranate	Y = 249,911X + 24,626	0.9965	11.5	0.02	97	4.7	9.3	11.5
					0.2	79	1.1	3.9	4.6
					0.4	84	3.1	4.0	5.4

**Table 2:** Dissipation rates (%) and half-lives of four pesticides in whole pomegranate based on SFO, DFOP and FOMC models.

Dissipation patterns	Parameters	Trial #1		Trial #2		Trial #5	
		PRO	DIF	PRO	DIF	PRO	DIF
SFO	DT50 (d)	11.7	9.07	12.9	12.3	9.6	8.56
	R^2^	0.9932	0.9791	0.9394	0.9947	0.9549	0.9469
	χ^2^ error (%)	3.82	8.17	11.3	3.15	13.2	13.7
DFOP	DT50 (d)	11.7	6.68	12.9	11.4	9.61	4.91
	R^2^	0.9932	0.9996	0.9395	0.9996	0.9549	0.9814
	χ^2^ error (%)	5.45	1.65	16.1	1.24	18.9	11.5
	DT50 (d)	11.7	7.1	12.8	11.3	9.6	6.1
FOMC	R^2^	0.9932	0.9967	0.9394	0.9992	0.9548	0.9691
	χ^2^ error (%)	4.37	3.59	12.9	1.34	15.1	11.8
7-day dissipation rate		36	51	29	37	27	58
14-day dissipation rate		53	66	38	57	56	63
21-day dissipation rate		70	76	71	68	87	76
28-day dissipation rate		85	85	87	78	93	91

## Data Availability

The original contributions presented in the study are included in the article/Supplementary Material, further inquiries can be directed to the corresponding author.

## Supplementary Materials

The following supporting information can be downloaded at: https://www.mdpi.com/article/10.3390/foods14223901/s1, Section S1. The preparation of standards. Section S2. Method validation. Figure S1. The structures of four pesticides and metabolite. Figure S2. Typical chromatograms of DIF(A), 2,4,6-trichlorophenol (B), SPI-A and-D (C), DIN, and metabolites (UF and DN) (D) standards (0. 1 mg/L). Table S1. Pomegranate cultivars, climate characteristics and soil physicochemical properties of trial sites across China. Table S2. Information on spray application parameters for difenoconazole, prochloraz, spinosad, and dinotefuran in pomegranates. Table S3. Retention time and mass spectrometry parameters of six analytes. Table S4. Pesticide residue concentrations (mg/kg) at different sampling intervals in trials#1, #2, and #5. Table S5. Crops registered for DIF and pro in China. Table S6. Non-carcinogenic effects assessment of PRO in pomegranates using deterministic and probabilistic models.

## Abbreviations

The following abbreviations are used in this manuscript:

DIF	Difenoconazole
PRO	Prochloraz
SPI	Spinosad
DIN	Dinotefuran
UF	UF (Metabolite of DIN)
DN	DN (Metabolite of DIN)
MRL	Maximum Residue Limit
ARfD	Acute Reference Dose
ADI	Acceptable Daily Intake
IESTI	International Estimated Short-Term Intake
NEDI	National Estimated Daily Intake
HQ	Hazard Quotient
RfD	Reference Dose
STMR	Supervised Trials Median Residue
HR	Highest Residue
UHPLC-MS/MS	Ultra-High Performance Liquid Chromatography-Tandem Mass Spectrometry
GC-ECD	Gas Chromatography with Electron Capture Detector
HPLC	High Performance Liquid Chromatography
QuEChERS	Quick, Easy, Cheap, Effective, Rugged, Safe
MRM	Multiple Reaction Monitoring
ESI	Electrospray Ionization
JMPR	Joint FAO/WHO Meeting on Pesticide Residues
OECD	Organisation for Economic Co-operation and Development
CAC	Codex Alimentarius Commission
FAO	Food and Agriculture Organization of the United Nations
PHI	Pre-Harvest Interval
LOQ	Limit of Quantification
ME	Matrix Effect
bw	Body Weight
SFO	Single First-Order
DFOP	Double First-Order in Parallel
FOMC	First-Order Multi-Compartment
