# Supplementary material for: Occurrence, Dissipation and Risk Assessment of Widespread Pesticides and Their Metabolites in Pomegranates
Source: Foods. 2025 Nov 14;14(22):3901. doi: 10.3390/foods14223901 (PMC12652290; doi:10.3390/foods14223901)
Supplement: Supplementary file 1 [file foods-14-03901-s001.zip › foods-3950792-supplementary.pdf]

*Article*

# Occurrence, dissipation and risk assessment of widespread pesticides and their metabolites in pomegranates

**Yuxiao Zhu [1,2,3], Rumei Li [1,2,3], Tongjin Liu [1,2,3], Ruijuan Li [1,2,3], Feng Fang [1,2,3*], Hui Liang [1,2,3*]**

[1] *Institute of Plant Protection, Shandong Academy of Agricultural Sciences, Jinan 250100, China;*
sdgtzyx@163.com (Y.Z.); rumeili0815@163.com(R.L.); tongjinliu2024@163.com (T.L.); ruijuanli@ 163.com(R.L.);
[2] *National Center for Agricultural Biosafety Sciences Shandong Sub-Center, Jinan 250100, China;*
sdgtzyx@163.com (Y.Z.); rumeili0815@163.com(R.L.); tongjinliu2024@163.com (T.L.); ruijuanli@ 163.com(R.L.)
[3] *Shandong Key Laboratory for Green Prevention and Control of Agricultural Pests, 250100, China*
sdgtzyx@163.com (Y.Z.); rumeili0815@163.com(R.L.); tongjinliu2024@163.com (T.L.); ruijuanli@ 163.com(R.L.)
* Correspondence: huiliangchem@163.com(H.L.), and weedfang@163.com(F.F.)

1. The preparation of standards .......... 3

2. Method validation .......... 3

Figure S1 The structures of four pesticides and metabolite .......... 4

Figure S2 Typical chromatograms of DIF(A), 2,4,6-trichlorophenol (B), SPI-A and-D (C), DIN, and metabolites (UF and DN) (D) standards (0. 1 mg/L), .......... 5

Table S1 Pomegranate cultivars, climate characteristics and soil physicochemical properties of trial sites across China .......... 5

Table S2. Information on spray application parameters for difenoconazole, prochloraz, spinosad, and dinotefuran in pomegranates. .......... 6

Table S3 Retention time and mass spectrometry parameters of six analytes .......... 7

Table S5 Crops registered for DIF and pro in China .......... 8

Table S6 Non-carcinogenic effects assessment of PRO in pomegranates using deterministic and probabilistic models .......... 10

1. The preparation of standards

The individual stock solutions of SPI-A, SPI-D, DIN, UF, and DN were prepared in acetonitrile, whereas 2,4,6-trichlorophenol was prepared in petroleum ether, all at a concentration of 1000 mg/L. All stock solutions were stored at -20°C in amber glass vials and were stable for at least 6 months. Working standard solutions were prepared by appropriate dilution of stock solutions with acetonitrile or petroleum ether to achieve the desired concentrations for calibration curves. For UHPLC-MS/MS analysis (DIF, SPI-A, SPI-D, DIN, UF, and DN), both solvent-based and matrix-matched calibration standards were prepared at concentrations of 0.01, 0.05, 0.1, 0.5, and 1.0 mg/L to establish calibration curves ranging from 0.01 to 1 mg/L. For GC-ECD analysis (2,4,6-trichlorophenol), solvent-based calibration standards were prepared in petroleum ether at the same concentration range. Matrix-matched standards (0.03-3.0 mg/L) were prepared by spiking appropriate volumes of working standard solutions into blank pomegranate extracts.

2. Method validation

The analytical method was validated to ensure its suitability for the intended purpose. Blank pomegranate samples (both arils and whole pomegranates) for method validation were obtained from the market in Shandong. Prior to use, the blank matrices were thoroughly analyzed using the developed analytical method to confirm the absence of target analytes and potential interfering compounds. The confirmed blank samples were homogenized and stored at -20°C until use in validation experiments Validation parameters included accuracy (mean recovery of spiked samples at three different spiking levels), precision (intra-day and inter-day repeatability expressed as percent relative standard deviation, %RSD), sensitivity (limit of quantitation (LOQ)), and linearity (or the linear range of measurement), and matrix effects. Calibration curves were constructed using both solvent-based standards and matrix-matched standards to evaluate linearity and matrix effects. Control measures were based on the outline in SANTE/12682/2019. The measurement uncertainty of the analytical method was assessed following the EURACHEM/CITAC guidelines.

The spiking procedure was as follows:

1. Blank matrix samples (10.0 ± 0.01 g) were accurately weighed into 50-mL centrifuge tubes.
2. Appropriate volumes of working standard solutions in acetonitrile (for DIF, SPI, DIN, UF, and DN) or petroleum ether (for PRO as 2,4,6-trichlorophenol) were added to achieve three fortification levels: DIF: 0.02 (LOQ), 0.1, and 1.0 mg/kg, PRO (as 2,4,6-trichlorophenol): 0.05 (LOQ), 0.5, and 7.0 mg/kg, SPI-A and D: 0.02 (LOQ), 0.3, and 1 mg/kg, DIN: 0.02 (LOQ), 0.2, and 0.4 mg/kg, UF: 0.02 (LOQ), 0.2, and 0.4 mg/kg, and DN: 0.02 (LOQ), 0.2, and 0.4 mg/kg.
3. After spiking, samples were thoroughly mixed by vortexing for 1 minute and allowed to stand at room temperature for 30 minutes to ensure complete solvent evaporation and equilibration of the analytes with the matrix.
4. The spiked samples were then processed according to the QuEChERS extraction and cleanup procedures described in Section 2.3 (Analytical procedures).
5. For intraday precision assessment, five replicate samples (n=5) were prepared and analyzed on the same day at each fortification level for both pomegranate arils and whole pomegranate matrices. For interday precision evaluation, three replicate samples (n=3) were prepared and analyzed on three different days at each fortification level.

6. Average recoveries, relative standard deviations (RSD), and expanded measurement uncertainty were calculated

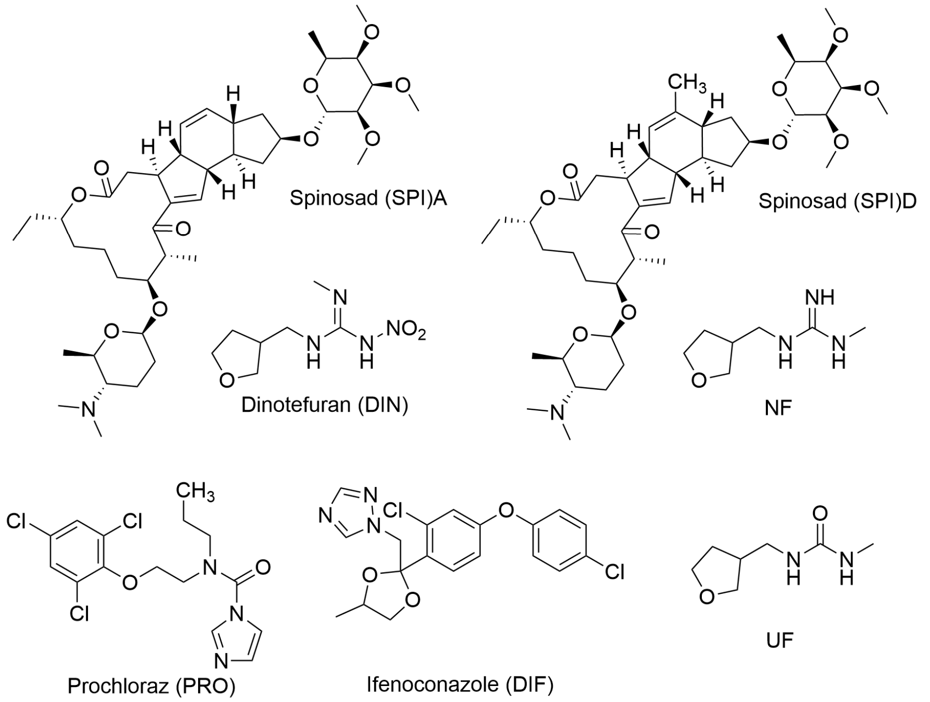


Fig. S1 The structures of four pesticides and metabolite

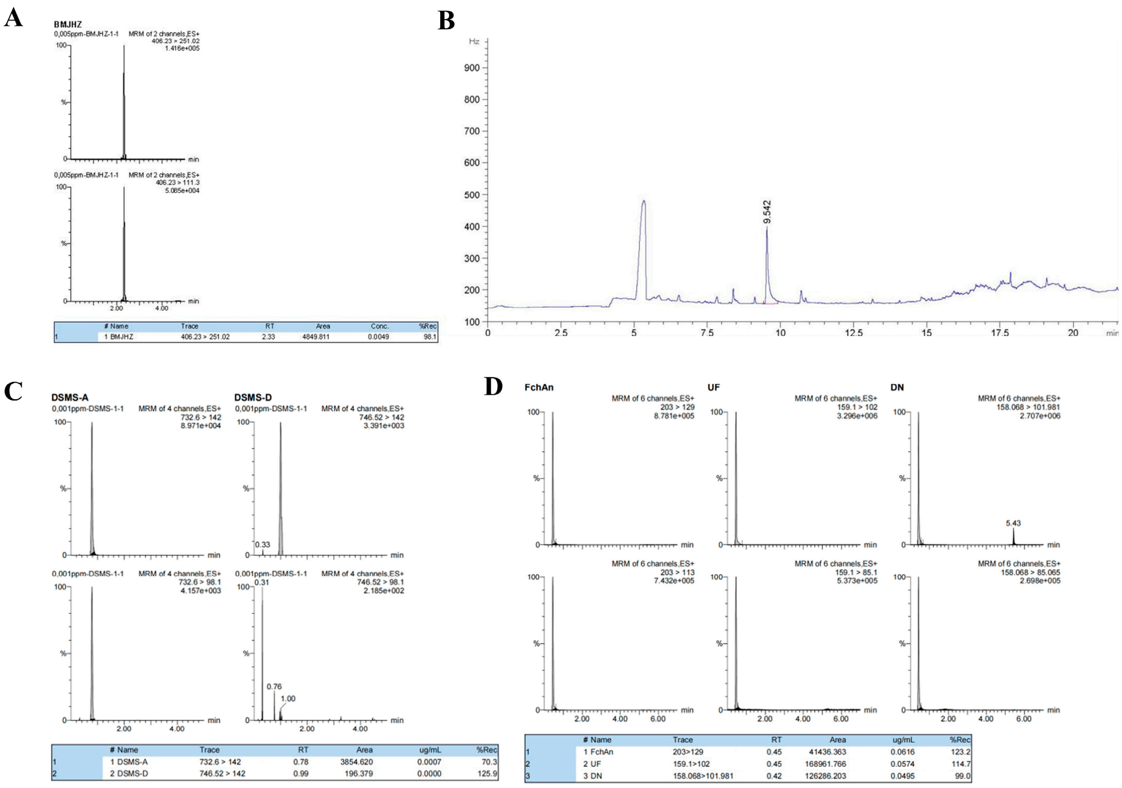

Fig. S2 Typical chromatograms of DIF(A), 2,4,6-trichlorophenol (B), SPI-A and-D (C), DIN, and metabolites (UF and DN) (D) standards (0. 1 mg/L),

Table S1 Pomegranate cultivars, climate characteristics and soil physicochemical properties of trial sites across China

| Trial Sites | Crop Cultivars | Climate Factors | | |
|---|---|---|---|---|
| | | Climate Type | Maximum and minimum temperatures | Cumulative Rainfall (mm) |
| Trial #1 Shandong | Hongpao pomegranate | Temperate monsoon climate | 19 ~ 36 ℃ | 17 |
| Trial #2 Shanxi | Hongpao pomegranate | Temperate continental monsoon climate | 20 ~ 33 ℃ | 9 |
| Trial #3 Henan | Hongpao pomegranate | Temperate continental monsoon climate | 16 ~ 37 ℃ | 5 |
| Trial #4 Hebei | Tunisian soft-seed pomegranate | Temperate continental monsoon climate | 8 ~ 34 ℃ | 56 |
| Trial #5 Anhui | Mengzi pomegranate | Humid subtropical climate | 21 ~ 37 ℃, | 86 |
| Trial #6 Yunnan | Honghua Yushizi pomegranate | Plateau mountain climate | 18 ~ 31 ℃ | 72 |

Table S2. Information on spray application parameters for difenoconazole, prochloraz, spinosad, and dinotefuran in pomegranates.

| | Difenoconazole | Prochloraz | Spinosad | Dinotefuran |
|---|---|---|---|---|
| Pesticide formulation | Water Dispersible Granule | Emulsion, Oil in Water | Suspension Concentrate | Suspension Concentrate |
| Dosage, mg a.i./kg | 100 | 450 | 62.5 | 100 |
| Spray times | 3 | 3 | 1 | 1 |
| Application interval, d | 10 | 10-14 | - | - |
| Pre-harvest intervals, d | 14 | 14 | 14 | 14 |

Table S3 Retention time and mass spectrometry parameters of six analytes

| Compound | Retention time (min) | Quantitative ion pair [a] (m/z) | Qualitative ion pair [b] (m/z) | Cone voltage (V) | Collision energy (eV) |
|---|---|---|---|---|---|
| DIF | 2.33 | 406.23/251.02 | 406.23/111.3 | 20 | 26<br>60 |
| SPI-A | 0.78 | 732.6/142 | 732.6/98.1 | 25 | 30<br>35 |
| SPI-D | 0.99 | 746.52/142 | 746.52/98.1 | 30 | 31<br>35 |
| DIN | 0.45 | 203/129 | 203/113 | 20 | 10<br>10 |
| UN | 0.45 | 159.1/102 | 159.1/85.1 | 15 | 10<br>16 |
| DF | 0.42 | 158.068/101.981 | 158.068/85.065 | 13 | 14<br>14 |

Table.S4 Pesticide residue concentrations (mg/kg) at different sampling intervals in trials#1, #2, and #5.

| Compouds | Sampling interval (d) | Concentration (mg/kg) | | |
|---|---|---|---|---|
| | | #1 | #2 | #5 |
| PRO | 0 | 0.86 | 0.78 | 1.2 |
| | 7 | 0.55 | 0.55 | 0.88 |
| | 14 | 0.40 | 0.48 | 0.53 |
| | 21 | 0.26 | 0.23 | 0.16 |
| | 28 | 0.13 | 0.098 | 0.079 |
| DIF | 0 | 0.41 | 0.30 | 0.72 |
| | 7 | 0.20 | 0.19 | 0.30 |
| | 14 | 0.14 | 0.13 | 0.27 |
| | 21 | 0.097 | 0.097 | 0.17 |
| | 28 | 0.060 | 0.066 | 0.065 |
| SPI | 0 | 0.047 | 0.021 | 0.18 |
| | 7 | -- | -- | -- |
| | 14 | -- | -- | -- |
| | 21 | -- | -- | -- |
| | 28 | -- | -- | -- |
| DIN | 0 | 0.34 | 0.13 | 0.41 |
| | 7 | 0.075 | 0.093 | 0.073 |
| | 14 | -- | -- | -- |
| | 21 | -- | -- | -- |
| | 28 | -- | -- | -- |

Table S5 Crops registered for DIF and pro in China

| Compound | Registered crop |
|---|---|
| DIF | Rice (Oryza sativa), Wheat (Triticum aestivum), Corn/Maize (Zea mays), Soybean (Glycine max), Potato (Solanum tuberosum), Chinese Yam (Dioscorea opposita), Tomato (Solanum lycopersicum), Chili Pepper (Capsicum annuum), Celery (Apium graveolens), Asparagus (Asparagus officinalis), Bitter Gourd (Momordica charantia), Zucchini (Cucurbita pepo), Cowpea (Vigna unguiculata), Loofah/Sponge Gourd (Luffa cylindrica), Common Bean (Phaseolus vulgaris), Winter Melon (Benincasa hispida), Chinese Toon (Toona sinensis), Chinese Cabbage (Brassica rapa subsp. pekinensis), Cucumber (Cucumis sativus), Onion (Allium cepa), Garlic Scapes (Allium sativum), Pumpkin (Cucurbita moschata), Apple (Malus domestica), Lychee (Litchi chinensis), Mango (Mangifera indica), Banana (Musa spp.), Jujube/Chinese Date (Ziziphus jujuba), Grape (Vitis vinifera), Hami Melon/Cantaloupe (Cucumis melo var. cantaloupensis), Dragon |

| | |
|---|---|
| | Fruit (Hylocereus undatus), Citrus (Citrus spp.), Cherry (Prunus avium), Plum (Prunus domestica), Loquat (Eriobotrya japonica), Lemon (Citrus limon), Persimmon (Diospyros kaki), Peach (Prunus persica), Pear (Pyrus spp.), Kiwi (Actinidia chinensis), Sweet Melon (Cucumis melo), Pomegranate (Punica granatum), Strawberry (Fragaria × ananassa), Watermelon (Citrullus lanatus), Kumquat (Citrus japonica), Sugarcane (Saccharum officinarum), Sesame (Sesamum indicum), Peanut (Arachis hypogaea), Chinese Walnut (Carya cathayensis), Hazelnut (Corylus avellana), Sunflower (Helianthus annuus), Cotton (Gossypium spp.), Tea Oil Camellia (Camellia oleifera), Tea (Camellia sinensis), Tobacco (Nicotiana tabacum), Vine Tea (Ampelopsis grossedentata), Honeysuckle (Lonicera japonica), Goji Berry (Lycium barbarum), Chrysanthemum (Chrysanthemum morifolium), Rose (Rosa spp.), Iron Skin Dendrobium (Dendrobium officinale), Panax Notoginseng (Panax notoginseng), Fritillaria (Fritillaria cirrhosa), Ginseng (Panax ginseng), Green Onion/Scallion (Allium fistulosum), Garlic (Allium sativum), Ginger (Zingiber officinale), Sichuan Peppercorn (Zanthoxylum piperitum). |
| PRO | Rice (*Oryza sativa*), Wheat (*Triticum aestivum*), Potato (*Solanum tuberosum*), Chinese Yam (*Dioscorea opposita*), Tomato (*Solanum lycopersicum*), Chili Pepper (*Capsicum annuum*), Celery (*Apium graveolens*), Cucumber (*Cucumis sativus*), Bitter Gourd (*Momordica charantia*), Loofah/Sponge Gourd (*Luffa cylindrica*), Garlic Scapes (*Allium sativum*), Shiitake Mushroom (*Lentinula edodes*), Water Bamboo (*Zizania latifolia*), Strawberry (*Fragaria × ananassa*), Lychee (*Litchi chinensis*), Chinese Bayberry (*Myrica rubra*), Jujube/Chinese Date (*Ziziphus jujuba*), Citrus (*Citrus* spp.), Pear (*Pyrus* spp.), Dragon Fruit (*Hylocereus undatus*), Mango (*Mangifera indica*), Grape (*Vitis vinifera*), Watermelon (*Citrullus lanatus*), Banana (*Musa* spp.), Apple (*Malus domestica*), Longan (*Dimocarpus longan*), Rapeseed/Canola (*Brassica napus*), Peanut (*Arachis hypogaea*), Garlic (*Allium sativum*), Ginger (*Zingiber officinale*), Iron Skin Dendrobium (*Dendrobium officinale*), Goji Berry (*Lycium barbarum*). |

Table S6 Non-carcinogenic effects assessment of PRO in pomegranates using deterministic and probabilistic models

| Gender | Age | Determination model (%) | | Probabilistic model (%) | | | | | | | |
|---|---|---|---|---|---|---|---|---|---|---|---|
| | | | | Urban | | | | Rural | | | |
| | | Urban | Rural | P50 | P75 | P95 | P99.9 | P50 | P75 | P95 | P99.9 |
| Male | 3-5 | 21.0 | 10.9 | 19.6 | 24.1 | 33.1 | 42.3 | 10.2 | 12.5 | 17.3 | 22.0 |
| | 6-11 | 13.1 | 10.5 | 12.3 | 15.1 | 20.8 | 26.5 | 9.8 | 12.1 | 16.6 | 21.2 |
| | 12-17 | 6.2 | 5.6 | 5.8 | 7.2 | 9.9 | 12.6 | 5.2 | 6.4 | 8.8 | 11.2 |
| | 18-59 | 4.2 | 2.5 | 3.9 | 4.8 | 6.6 | 8.4 | 2.3 | 2.8 | 3.9 | 5.0 |
| | >60 | 5.0 | 2.1 | 4.7 | 5.8 | 8.0 | 10.1 | 2.0 | 2.4 | 3.4 | 4.3 |
| Female | 3-5 | 15.4 | 14.2 | 14.4 | 17.7 | 24.4 | 31.1 | 13.3 | 16.3 | 22.5 | 28.6 |
| | 6-11 | 15.0 | 12.3 | 14.0 | 17.2 | 23.7 | 30.2 | 11.5 | 14.1 | 19.5 | 24.8 |
| | 12-17 | 7.7 | 7.4 | 7.2 | 8.8 | 12.2 | 15.5 | 6.9 | 8.5 | 11.7 | 15.0 |
| | 18-59 | 6.8 | 3.7 | 6.4 | 7.8 | 10.8 | 13.7 | 3.5 | 4.3 | 5.9 | 7.5 |
| | >60 | 6.4 | 2.7 | 6.0 | 7.4 | 10.2 | 13.0 | 2.5 | 3.0 | 4.2 | 5.4 |
